# Enhanced Photocatalytic Activity of V_2_C MXene-Coupled ZnO Porous Nanosheets with Increased Surface Area and Effective Charge Transfer

**DOI:** 10.3390/ma17112529

**Published:** 2024-05-24

**Authors:** Weibing Zhou, Lilong Sun, Kang Li, Shouqin Tian

**Affiliations:** 1 School of Materials Science and Engineering, Wuhan University of Technology, Wuhan 430070, China; jsyczwb@whut.edu.cn (W.Z.); 331073@whut.edu.cn (L.S.); likang1803@whut.edu.cn (K.L.); 2 State Key Laboratory of Silicate Materials for Architectures, Wuhan University of Technology, Wuhan 430070, China

**Keywords:** porous ZnO nanosheets, V_2_C MXene, photothermal effects, photocatalysis

## Abstract

Photocatalysis performs excellently when degrading organic pollutants, but the photocatalytic degradation rate is not high for most photocatalysts due to their narrow sunlight adsorption range and high recombination rate of electron hole pairs. Herein, we use V_2_C-MXene with a wide sunlight adsorption range to couple ZnO porous nanosheets and form ZnO/MXene hybrids using a facile electrostatic self-assembly method. The ZnO/MXene hybrids acquired demonstrated improved photochemical efficiency in breaking down methylene blue (MB) when contrasted with porous ZnO nanosheets. The degradation rate of MB reached 99.8% under UV irradiation for 120 min after the ZnO/MXene hybrid formation, while 38.6% was attained by the ZnO porous nanosheets. Moreover, photodegradation rate constants (k) were calculated as 3.05 × 10^−3^ and 5.42 × 10^−2^ min^−1^ for ZnO porous nanosheets and ZnO/MXene hybrids, respectively, indicating that the photodegradation performance was enhanced by 17.8 times after the modification of V_2_C. This was probably because the modification of V_2_C can increase the specific surface area to provide more sites for MB adsorption, widen the sunlight adsorption range to produce good photothermal effect, and facilitate the transfer of photogenerated carriers in ZnO to promote the reaction of more photogenerated carriers with MB. Hence, this work offers a simple approach to creating effective photocatalysts for breaking down organic contaminants.

## 1. Introduction

In recent years, water pollution has become more and more serious, with the rapid industrial development, including textiles, printing, and dyeing, seriously affecting the human living environment and ecological security [1,2]. In this sense, the purification of sewage is particularly important. Traditional sewage treatment methods include adsorption [3,4,5], membrane separation [6,7], photocatalysis [8,9], and other advanced oxidation processes (AOP) [10,11]. Among these methods, semiconductor photocatalysis has attracted widespread attention due to its high efficiency, environmental friendliness, and ease of operation [12].

Zinc oxide is a semiconductor material with a bandgap of approximately 3.37 eV [13,14]. In the field of photocatalysis, its band gap is similar to that of traditional TiO_2_, and ZnO is favored because of its non-toxic, excellent photoelectric chemical stability, low cost, rich morphology, and higher photocatalytic activity. It is considered to be the most ideal material to replace TiO_2_ [15,16,17]. However, there are few active sites on the surface of ZnO and it only absorbs UV light. Moreover, the rapid recombination rate of electron hole pairs produced by light decreases their rate of degradation. In order to improve the photocatalytic efficiency of ZnO, co-catalysts are one of the most effective methods, such as metal deposition [18,19], heterojunction construction [20], and co-catalysts [21,22].

Recently, a new two-dimensional nanomaterial called MXene has been proposed as an effective co-catalyst because of its special morphology, exposed metal potential, and good electrical conductivity [23,24,25]. MXene has been successfully applied as a co-catalyst in TiO_2_ [26], BiOCl [27], g-C_3_N_4_ [28], and these composites exhibited better photocatalytic performance. ZnO/MXene hybrids with a graded flower-like structure in our previous work [29] also showed excellent photocatalytic activity due to 2D/2D heterostructure with efficient charge transfer. The ZnO nanosheets were obtained by using zinc acetate as the zinc source with a large thickness. In order to form better 2D/2D heterostructure, the thickness of ZnO nanosheets should be reduced. Due to the differences in the morphology of ZnO synthesized from different zinc sources, ZnO nanosheets can be synthesized from zinc sulfate as the zinc source and exhibit a thinner lamellar structure [30], with their dimensions being closer to those of V_2_C MXene and able to form a tightly coupled structure; therefore, ZnO/MXene will show a higher catalytic efficiency. Therefore, it is of great necessity to obtain better 2D/2D heterostructure between ZnO nanosheets and MXene with effective charge transfer for enhanced photocatalytic performance.

In this work, ZnO porous nanosheets with a thin thickness of 35 nm were obtained by using zinc sulfate as the zinc source, and then ZnO/MXene nanocomposites were prepared by combining ZnO porous nanosheets with 2D V_2_C MXene through facile electrostatic self-assembly. In addition, under ultraviolet light, the photocatalytic degradation performance of ZnO/MXene composites for MB was investigated. The results show that the photocatalytic properties of MB are better than those of ZnO porous nanosheets. The mechanism for enhancing the photocatalytic properties of MB is discussed.

## 2. Experimental Section

### 2.1. Materials

The reagents vanadium carbide M_n+1_AX_n_ (V_2_AlC) was purchased from Nanjing Mingchang New Materials Technology Co., Ltd. (Nanjing, China). tetramethylammonium hydroxide (TMAH, C_4_H_13_NO), zinc sulfate heptahydrate (ZnSO_4_·7H_2_O), hexamethylenetetramine (HMTA, C_6_H_12_N_4_), hydrofluoric acid (HF), and methylene blue (MB, C_16_H_18_ClN_3_S) were used, which were purchased from China National Pharmaceutical Group Chemical Reagent Co., Ltd. (Beijing, China). The reagents used are analytical reagents that can be used directly without purification. Deionized water was used as test water.

### 2.2. Preparation of ZnO/MXene Composite

**Preparation of porous ZnO nanosheets**: a total of 0.02 moles of zinc sulfate (ZnSO_4_) and 0.02 moles of hexamethylenetetramine (HMTA) was separately dissolved in 30 mL of deionized water. The solutions were then stirred at room temperature until they became homogeneous. Then, under the action of a magnetic field, the HMTA mixture was added to the zinc sulfate solution. After that, the mixed solution was transferred to a reactor for hydrothermal reactions at 150 °C for 12 h. The precipitate obtained after the reaction was washed and dried to obtain white powders. Finally, the white powder was calcined at 500 °C for 5 h to obtain 1.38 g of ZnO nanosheets.

**Preparation of V_2_C MXene**: the V_2_C MXene was synthesized through a top-down method. Initially, 4 g of V_2_AlC MAX powders, sized using a 500 mesh, were gently submerged in 50 milliliters of a 49% hydrofluoric acid solution and agitated at 60 °C for a period of 48 h. Then, the filtered suspension was washed and filtered with deionized water until the pH reached approximately 6. Afterward, the obtained precipitate was dried under vacuum at 60 °C and the dried sample was dispersed with TMAH at a mole ratio of 1:10 for layering treatment and the TMAH was then removed by washing with deionized water. Finally, 1.53 g V_2_C MXene was obtained after drying the sample under vacuum at 60 °C.

**Figure 1:**
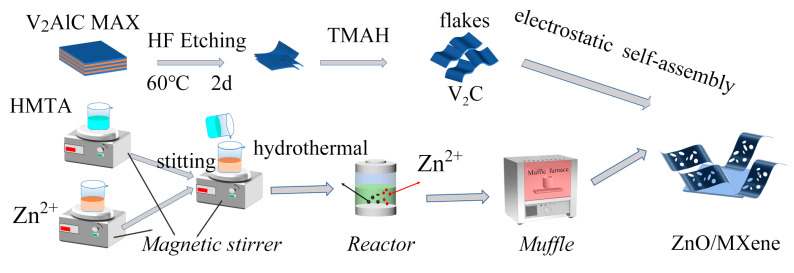
Schematic diagram of the preparation process for the ZnO/MXene hybrids.

**Preparation of ZnO/MXene composite:** the composite of ZnO/MXene was synthesized through an electrostatic self-assembly technique. Firstly, 0.98 g porous ZnO nanosheets were dispersed in 50 mL of deionized water and stirred with magnetic force for 0.5 h. Subsequently, 0.02 g layered V_2_C MXene was added and stirring was continued for 0.5 h (the mass percentage was 2%). Then, the mixed solution continued to be stirred for 3 h to obtain 2%V_2_C–98%ZnO composite. Finally, it was dried at 60 °C to obtain 0.99 g of ZnO/MXene composites. The detailed experimental process is illustrated in Figure 1.

### 2.3. Characterizations

The phase structure of the sample was analyzed using XRD (Empyrean, Almere, The Netherlands), while the morphology was examined using scanning electron microscopy (SEM, JSM-IT800, Kyoto, Japan). Further analysis of the microstructures and elemental distributions of ZnO/MXene composites was carried out utilizing the transmission electron microscope (TEM, Talos F200S, Waltham, MA, USA). The chemical composition and valence states of the sample were studied with X-ray photoelectron spectroscopy (XPS, Escalab 250Xi, Waltham, MA, USA). A UV vis absorption spectrophotometer (DRS, UV-2600, Kyoto, Japan) was used to obtain diffuse reflectance spectra in the wavelength range of 200 nm to 800 nm, with BaSO_4_ powder acting as the reference material. The specific surface area and pore size distribution were determined using an N_2_ adsorption instrument (BET, ASAP 2020, Norcross, GA, USA).

### 2.4. Photocatalytic Activity of the ZnO/MXene Composite

In order to assess the photocatalytic performance of the ZnO/MXene composite, the degradation of methylene blue under photocatalysis was carried out at a starting concentration of 1.0 × 10^−5^ mol/L. The trial was executed under ambient conditions with a xenon lamp (CHF-XM, 500 W) utilized as the source of ultraviolet radiation. The experimental procedure was conducted in the following manner. Initially, a 50 mg quantity of ZnO/MXene photocatalyst was dispersed into a 10 mL solution of MB, which was then left in the dark while being stirred magnetically for 30 min to establish an equilibrium between the MB molecules and the ZnO/MXene photocatalyst. Subsequently, the suspension underwent exposure to ultraviolet light for the photocatalytic degradation experiments. At specific time intervals of 20 min during the photodegradation process, 4 mL of the suspension was extracted and the photocatalyst was separated from the suspension using centrifugation. After that, the residual concentration of MB in the obtained supernatant following centrifugation was determined by absorption spectra at a wavelength of 664 nm. The calculation formula for the photodegradation rate of MB solution is shown below:

(1) $$Degredation\left(\%\right)=\frac{A_{0}-A}{A_{0}}\times 100\%$$

Among them, A_0_ and A are the initial absorbance and residual absorbance of MB in the solution, respectively. 

## 3. Results and Discussion

### 3.1. Structure and Morphology

**Figure 2:**
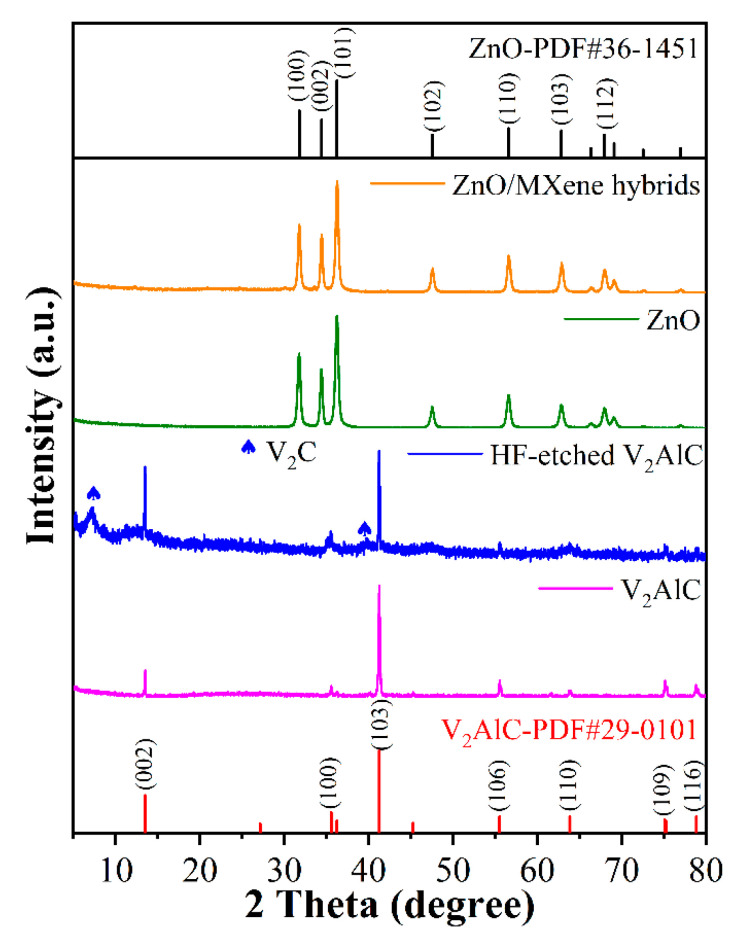
XRD patterns of MXene, ZnO, and ZnO/MXene composites.

The phase structure of the obtained samples was characterized by XRD and the result is shown in Figure 2. It can be seen that a new broad diffraction peak was observed at 2θ of 7.3° in the sample of HF-etched V_2_AlC compared with the V_2_AlC untreated, corresponding to the (002) crystal plane of V_2_C MXene, a c-lattice parameter (c-LP) of 24.4 Å. This indicated that partial V_2_AlC had been successfully converted to V_2_C MXene. In addition, there was a V_2_AlC MAX phase (PDF#29-0101) which remained in the HF-etched V_2_AlC, a phenomenon which is consistent with previous reports on the synthesis of V_2_CT_x_ [31]. On the other hand, the other diffraction peaks belonged to the hexagonal wurtzite structure of ZnO (PDF#36-1451), where the (002) facet is a polar surface and the (100) facet is a non-polar surface, suggesting that ZnO was present in the obtained composites and that the catalytic activity of the polar surface is superior to that of the non-polar surface. The diffraction peak intensity of the (100) and (002) crystal planes is related to the preferred orientation growth of the sample [32]. Moreover, only the ZnO phase was observed after forming the ZnO/MXene composite, with almost no diffraction peaks originating from MXene. This was because the amount of MXene in the ZnO/MXene composite was too small (2 wt%) and the detected diffraction peak signal was too weak to be observed [33].

**Figure 3:**
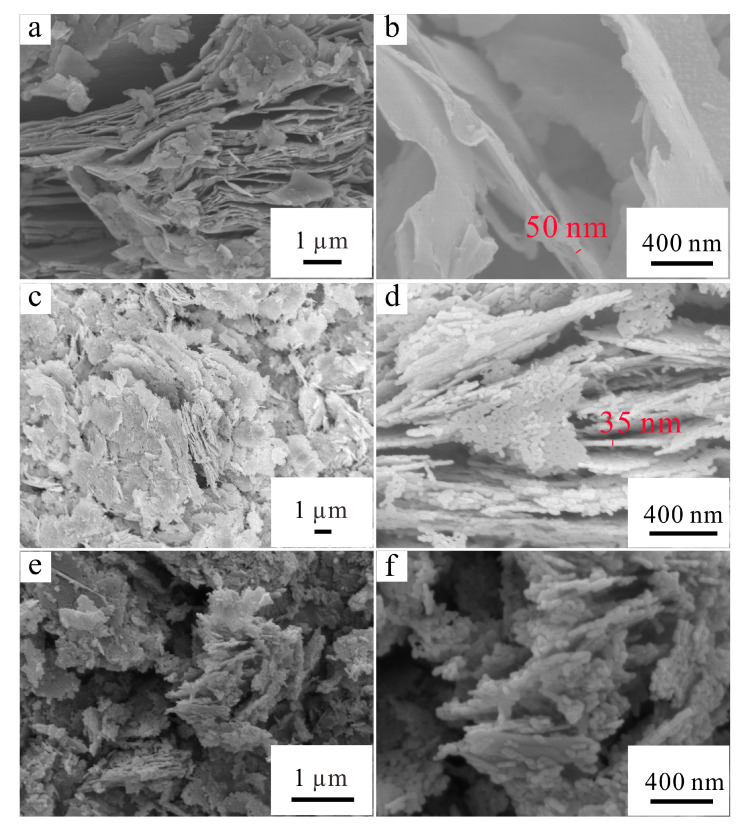
SEM images of HF-etched V_2_AlC (**a**,**b**), porous ZnO nanosheets (**c**,**d**), and ZnO/MXene composites (**e**,**f**).

In order to observe the morphology of HF-etched V_2_AlC, ZnO, and ZnO/MXene composites, SEM characterization was carried out and the result is shown in Figure 3. The SEM images of the V_2_C MXene in Figure 3a,b were obtained from HF-etched V_2_AlC. It is known from previous research that the V_2_AlC MAX without layering shows the shape of an accordion [34]. However, it exhibited 2D nanosheets with a thickness of ~50 nm after etching with the HF solution, showing increased spacing between layers (Figure 3a,b). On the other hand, many porous nanosheet structures formed by the self-assembly of a large number of ZnO nanoparticles were observed in the prepared ZnO sample, and the thickness of the nanosheets was about 35 nm (Figure 3c,d). It has been shown that the porous structure can increase the active site, which can absorb more organics during the photocatalytic process, thus effectively improving the photocatalytic efficiency [35]. Moreover, it can be seen from Figure 3e,f that the assembly of porous ZnO nanosheets with 2D MXene resulted in a better dispersion of ZnO nanosheets, a phenomenon which is conducive to better photocatalytic performance.

**Figure 4:**
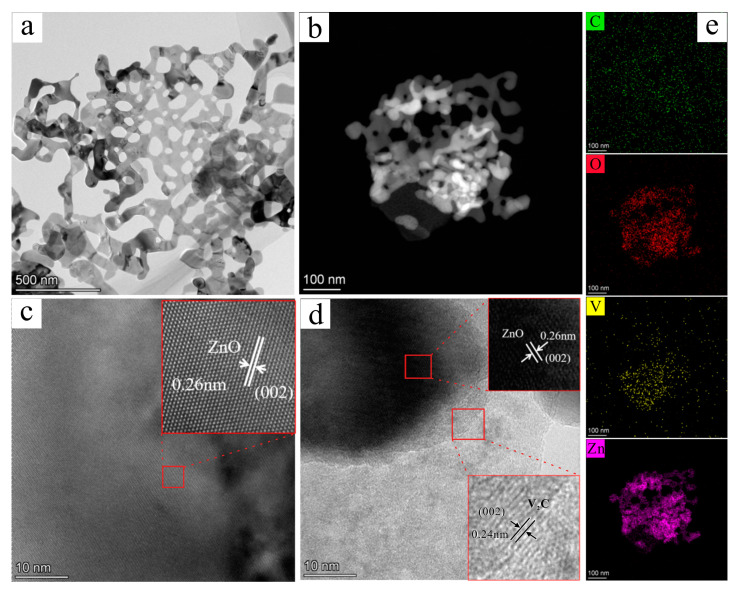
(**a**,**b**) TEM and (**c**,**d**) HRTEM images of porous ZnO nanosheets (**a**,**c**) and ZnO/MXene hybrids (**b**,**d**), and (**e**) elemental mapping of the ZnO/MXene composite.

In order to further analyze the microstructure of the samples, TEM characterization was conducted, and the findings are illustrated in Figure 4. In Figure 4a,b, it can be seen that a porous nanosheet was observed both in the ZnO sample and the ZnO/MXene composite, and the HRTEM image in Figure 4c revealed an interplanar spacing of 0.26 nm which aligned with the (002) crystal plane of ZnO. In addition, in the ZnO/V_2_C-MXene composites, there were two lattice fringes with lattice spacings of 0.26 nm and 0.24 nm, respectively, belonging to the (002) crystal plane of ZnO and the (002) crystal plane of V_2_C MXene (Figure 4d). This is consistent with the V_2_C MXene in XRD at 2θ and the peak appearing at 7.3°. In addition, Figure 4e illustrates the consistent dispersion of elements C, O, Zn, and V, providing evidence of an interface formation between ZnO and V_2_C MXene, as well as indicating the existence of potent interaction forces.

**Figure 5:**
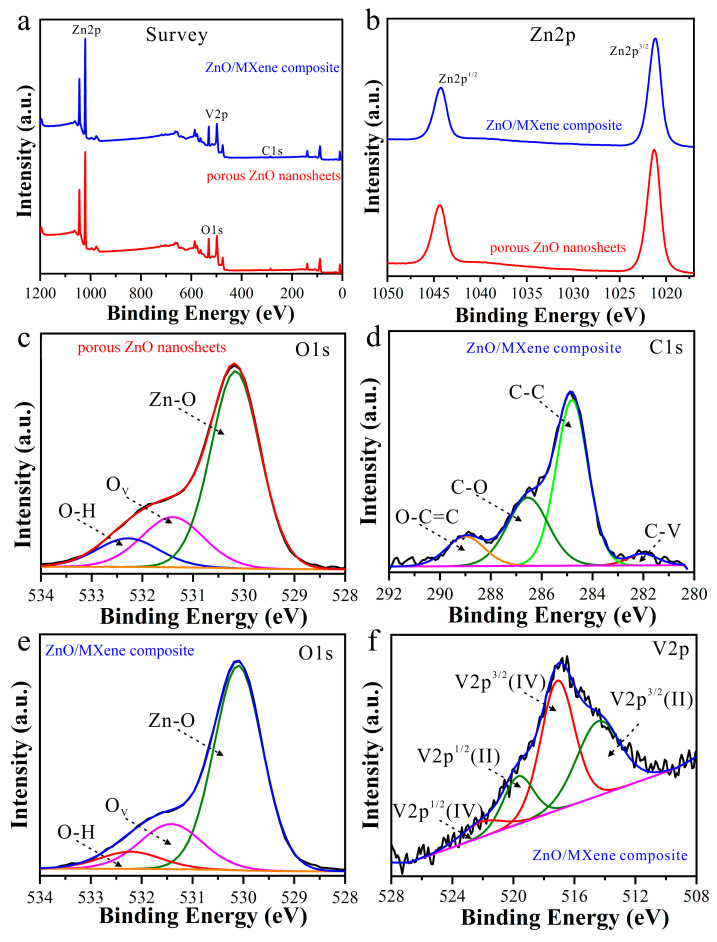
(**a**) Full XPS survey and (**b**) high-resolution Zn 2p spectra of porous ZnO nanosheets and ZnO/MXene composites, and XPS high-resolution O 1s spectra for (**c**) porous ZnO nanosheets. XPS high-resolution (**d**) C 1s, (**e**) O 1s and (**f**) V 2p spectra for the ZnO/MXene composite.

XPS characterization was conducted to further investigate the chemical composition and surface structures of the ZnO/MXene composite. The findings are depicted in Figure 5. It can be seen from the survey spectra (Figure 5a) that the prepared porous ZnO nanosheets consisted of Zn and O elements and the ZnO/MXene composite was mainly composed of Zn, V, C, and O elements. In addition, high-resolution Zn2p XPS spectra (Figure 5b) showed two peaks centered at ~1044.3 eV and ~1021.3 eV, corresponding to Zn2p^1/2^ and Zn2p^3/2^, respectively [36]. Figure 5c,e shows O 1s spectra of porous ZnO nanosheets and ZnO/V_2_C MXene composites which can be fitted onto three peaks, namely OH, oxygen vacancies (O_V_), and Zn–O bonds with binding energies of ~532.3 eV, ~531.3 eV, and ~530.4 eV, respectively [30], indicating that oxygen defects existed in both prepared porous ZnO nanosheets and ZnO/MXene composites. Moreover, the XPS high-resolution C1s spectrum of the ZnO/MXene composite in Figure 5d was deconvoluted into four peaks centered at ~282.1 eV, ~284.8 eV, ~286.1 eV, and ~289.0 eV, assigned to C–V, C–C, C–O, and O–C=C, respectively. Furthermore, Figure 5f shows the high-resolution V 2p spectra of the ZnO/MXene composite. The two peaks observed at 517.1 eV and 522.2 eV were identified as V^4+^ 2p^3/2^ and V^4+^ 2p^1/2^, respectively, while the additional peaks at 514.3 eV and 519.6 eV corresponded to V^2+^ 2p^3/2^ and V^2+^ 2p^1/2^, respectively. The presence of a monolayer of vanadium oxide on the surface of V_2_C was attributed to the V^4+^ peaks, whereas the formation of V–O bonds resulting from the interaction between V_2_C MXene and ZnO was suggested by the V^2+^ peaks [37]. In this sense, the MXene-modified porous ZnO nanosheets were successfully synthesized. ZnO and V_2_C form a heterojunction that provides a unique reaction interface. Meanwhile, ZnO acts as a donor of electrons which can be rapidly transferred to the V_2_C surface. In ZnO/MXene composites, the photogenerated carriers are mainly realized through the photogenerated charge separation and transfer process. When ZnO absorbs photons and generates photogenerated carriers, these electrons and holes are separated and the electrons are transferred to V_2_C while the holes remain in ZnO. In this process, the interface plays a very important role. The interface provides an efficient electron-transport channel so that the photogenerated electrons can be transported quickly and efficiently from ZnO to the MXene surface.

**Figure 6:**
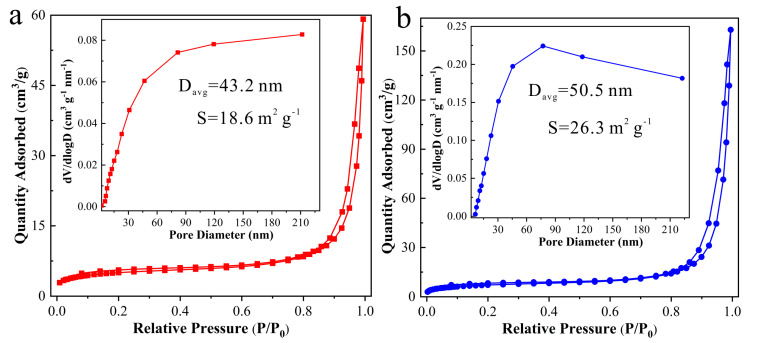
N_2_ adsorption-desorption isotherms and pore size distribution curves of (**a**) porous ZnO nanosheets and (**b**) ZnO/MXene composites.

To analyze the variance in surface area and distribution of pore sizes between porous ZnO nanosheets and ZnO/MXene composites, the N_2_ physical absorption and desorption isotherms were employed through the Brunauer–Emmett–Teller (BET) method and the results are shown in Figure 6. It can be seen that both porous ZnO nanosheets and ZnO/MXene composites showed type IV isotherms, indicating their porous structures [38]. Additionally, the specific surface area of the porous ZnO nanosheets and ZnO/MXene composite were 18.6 m^2^ g^−1^ and 26.3 m^2^ g^−1^, respectively, suggesting a higher surface area in the ZnO/MXene composite than in the ZnO nanosheets. Moreover, the pore size distribution curves (insets of Figure 6) indicate that both mesopores and macropores were observed in the porous ZnO nanosheets and ZnO/MXene composites, and their average pore size was 43.2 nm and 50.5 nm, respectively. A larger specific surface area can provide more active sites to facilitate catalysis [39]. This suggests that the ZnO/MXene composite with a higher surface area would exhibit better photocatalytic performances.

### 3.2. Photocatalytic Performance of ZnO/MXene Composite

**Figure 7:**
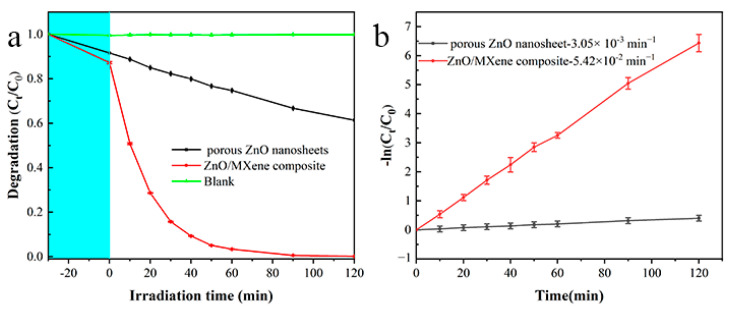
(**a**) Photocatalytic degradation curves and (**b**) degradation kinetics of porous ZnO nanosheets and ZnO/MXene composites.

Results of the MB photodegradation using the porous ZnO nanosheets and the ZnO/MXene composite as photocatalysts under the same operating conditions are shown in Figure 7. Prior to the photocatalysis, MB solutions with/without photocatalysts were treated in a dark environment for half an hour to ensure that the photocatalyst for the uptake of MB reached the absorption–desorption equilibrium. It can be seen that the adsorption amounts of MB for the porous ZnO nanosheets and ZnO/MXene composite were 10% and 13%, respectively (Figure 7a). Additionally, the concentration of MB remained almost unchanged under ultraviolet light irradiation in the absence of photocatalysts, indicating that hardly any decomposition of MB under the exposure to UV light had occurred. Moreover, an obvious degradation of MB was observed after the addition of photocatalysts. Under the condition of 120 min of UV irradiation, 38.6% MB was degraded in relation to the porous ZnO nanosheets, while the degradation proportion of MB reached 99.8% after the porous ZnO nanosheets had been combined with V_2_C MXene.

To assess the photocatalytic efficiency of the ZnO/MXene composite photocatalyst, the Langmuir–Hinshelwood (L–H) model was employed to analyze the apparent rate constant *k* for the degradation of organic dyes. The results are presented below:

(2) $$-\ln\left(\frac{C_{t}}{C_{0}}\right)=kt\;$$

*C*_0_ represents the MB concentration post treatment with dark absorption, where *k* and *t* stand for the degradation rate constant and the time of irradiation, respectively. The photodegradation rate constants were calculated as 3.05 × 10^−3^ min^−1^ and 5.42 × 10^−2^ min^−1^ for porous ZnO nanosheets and ZnO/MXene composites, respectively (Figure 7b). These results indicate that MXene-modified porous ZnO nanosheets have a higher photocatalytic activity, whose photodegradation rate constant was 17.8 times that of the porous ZnO nanosheets. Furthermore, compared with the other works on ZnO-based photocatalysts in Table 1, the obtained MXene-modified porous ZnO nanosheets exhibited excellent photodegradation activity.

**Figure 8:**
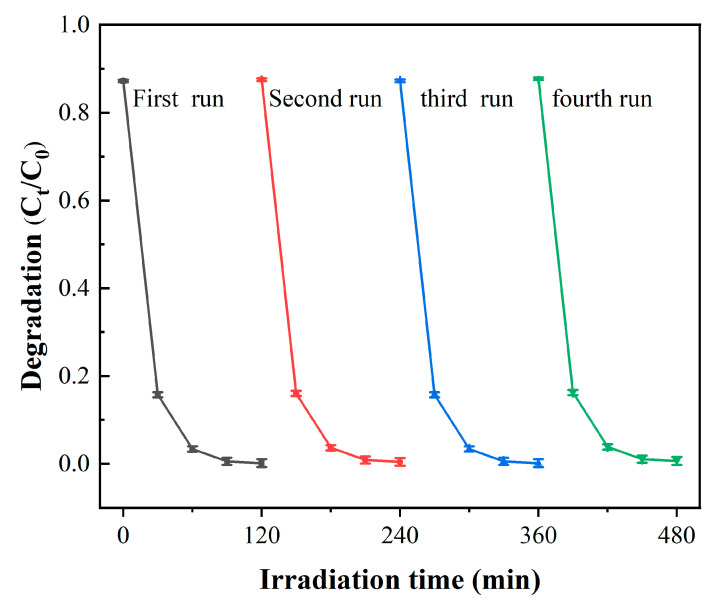
Cyclic test of the ZnO/MXene composite as a photocatalyst for photocatalytic degradation of MB.

On the other hand, reusability is necessary for the practical application of all photocatalysts in wastewater treatment. The result for the ZnO/MXene composite of the MB photocatalytic degradation stability is shown in Figure 8. Research has shown that, even after four cycles, the catalytic performance of the ZnO/MXene composite remains outstanding.

ToF (turnover frequency) is one of the most important indices for evaluating the photocatalytic reaction rate, which indicates the number of turnovers per unit time. The higher the photocatalytic TOF, the higher the catalytic efficiency and the faster the reaction speed. The degradation efficiency of ZnO reached 17.7% in 30 min, and the TOF of ZnO was calculated to be 0.0048 min^−1^, while the degradation efficiency of ZnO/MXene reached 84.3% in 30 min, and, thus, the TOF reached 0.055 min^−1^, which was 11.46 times higher than that of ZnO.

### 3.3. Possible Photocatalytic Mechanism

**Figure 9:**
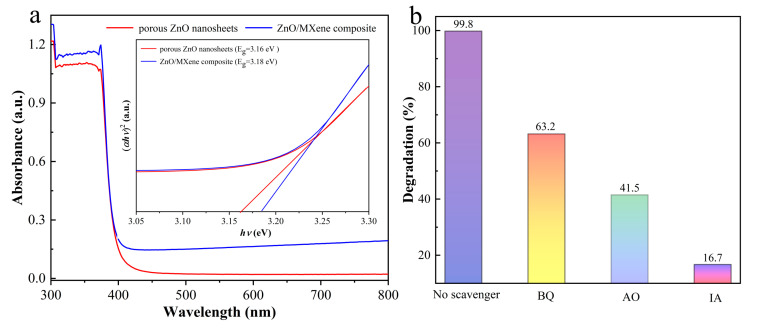
(**a**) DRS spectra and plots of (αhυ)^2^ vs. hυ (inset) of porous ZnO nanosheets and of the ZnO/MXene composite. (**b**) The variation in MB concentration under illumination for the ZnO/MXene composite after adding IA, AO, and BQ as free radical scavengers.

To further expound the possible photocatalytic mechanism of porous ZnO nanosheets and of ZnO/MXene composites, their light harvesting ability was investigated. Figure 9a shows the UV visible diffuse reflectance spectra. It can be seen that the optical band gap of porous ZnO nanosheets and of ZnO/MXene composites was similar and they were 3.16 eV and 3.18 eV, respectively. Although the addition of the V_2_C MXene to the porous ZnO nanosheets to form the ZnO/MXene composite cannot enlarge the absorption range of ZnO to visible light, as a photothermal material, it can promote photocatalysis by converting infrared light into heat energy through the photothermal effect [46].

As for the mechanism enhancing the photodegradation activity of ZnO/MXene composites, the following empirical equations can be used to calculate the conduction band and forbidden band of ZnO:

(3) $$E_{VB}=\chi -E_{e}+0.5E_{g}\;$$

(4) $$E_{CB}=E_{VB}-E_{g}\;$$

Here, *χ*, *E_CB_*, *E_VB_*, *E_e_*, and *E_g_* represent the electronegativity, conduction band energy, valence band energy, hydrogen scale-free electron energy (~4.5 eV), and forbidden band of the semiconductor, respectively. The absolute electronegativity *E_CB_* and *E_VB_* of ZnO were calculated to be 5.79, −0.28 V, and 2.88 V, respectively.

Moreover, in order to study the degradation mechanism of MB, the free radical capture experiment was carried out. Here, isopropanol (IA), ammonium oxalate (AO), and p-benzoquinone (BQ) were used as radical scavengers for ^•^OH^−^, h^+^, and ^•^O_2_^−^, respectively. The experimental results are shown in Figure 9b. The results show that all three kinds of trapping agents exerted influence on the photocatalytic process. It can be seen that, following the addition of IA, the photocatalytic efficiency became greatly reduced, indicating that the degradation of MB was mainly due to ^•^OH^−^, followed by h^+^ and ^•^O_2_^−^, a finding which is consistent with the previous results [47,48].

**Figure 10:**
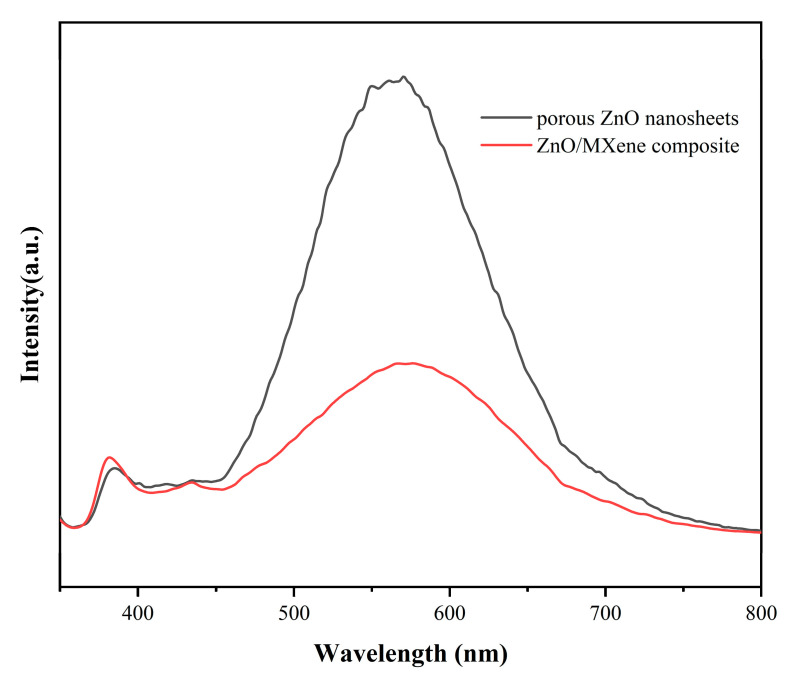
Photoluminescence spectroscopy of porous ZnO nanosheets and ZnO/MXene composites.

Figure 10 shows the photoluminescence spectroscopy (PL) results for both porous ZnO nanosheets and ZnO/MXene composites, demonstrating the effectiveness of conducting charge carrier separation and transfer analysis using this technique. The PL strength of ZnO/MXene composites was lower than that of porous ZnO nanosheets, and the composites with low PL strength exhibited a lower electron–hole recombination rate, which prolongs the lifetime of the carriers and is conducive to the improvement of photocatalytic activity [49]. At the excitation wavelength of 325 nm, the PL emission spectra of pure ZnO nanosheets and ZnO/MXene composites were located in the same position, indicating that the surface state of the samples was consistent. Due to the addition of V_2_C MXene, the transfer of photogenerated electrons was effectively promoted, the recombination between photogenerated carriers was inhibited, and the tight coupling interface formed also accelerated the charge transfer and inhibited the photogenerated electron recombination.

**Figure 11:**
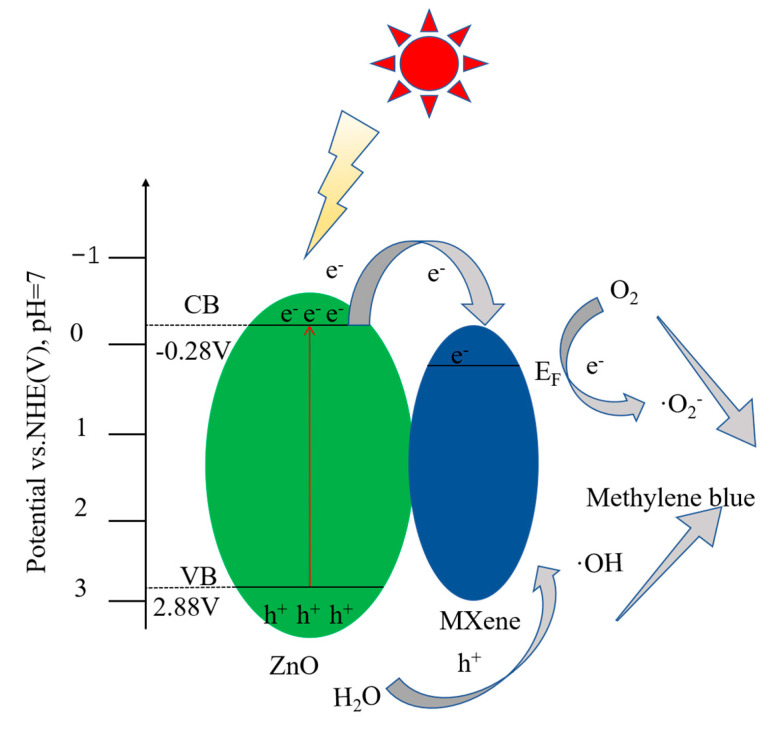
Mechanism diagram of photocatalytic degradation of MB for the ZnO/V_2_C hybrids.

According to the findings presented above, a potential photocatalytic mechanism has been suggested in order to elucidate the improved performance of ZnO/MXene composites in the photodegradation of organic dyes (Figure 11). For porous ZnO nanosheets under irradiation with sufficient high-energy light, the electron–hole pairs were generated and underwent oxidation–reduction to degrade dyes. Due to the high carrier recombination rate, porous ZnO nanosheets are not sensitive to MB degradation. On the other hand, due to the strong interaction interface between ZnO and V_2_C MXene, which is a photothermal material in ZnO/MXene composites, the latter can quickly transfer the photo generated electrons of ZnO to the Feimi level of V_2_C MXene, thereby reducing the electron recombination rate of ZnO. Moreover, the ZnO/MXene composite possessed higher specific surface areas, providing more active sites for organic dyes, further enhancing its photocatalytic activity. Regarding the MB degradation pathway, we can analyze the dyes in the degradation process, and we will further discuss the specific mechanism in future work.

## 4. Conclusions

In summary, V_2_C-MXene-coupled porous ZnO nanosheets (ZnO/Mxene hybrids) were successfully prepared by using an electrostatic self-assembly method and exhibited excellent photocatalytic degradation performance in degrading methylene blue compared with porous ZnO nanosheets, with their apparent rate constant being 17.8 times higher than that of the porous ZnO nanosheets. This is probably because the ZnO porous nanosheets were prepared with zinc sulfate as the zinc source and showed a larger specific surface area and thinner thickness than the ZnO nanosheets obtained with zinc acetate as the zinc source in our previous work. The thickness of the thinner ZnO nanosheets is closer to the thickness of V_2_C MXene, so that ZnO nanosheets were tightly combined with V_2_C MXene to form a tighter heterojunction structure. In ZnO/MXene hybrids, V_2_C served as a photothermal material and quickly transferred photogenerated electrons from the valence band of ZnO to the surface of V_2_C MXene, accelerating electron transfer and reducing its recombination rate. Furthermore, the larger specific surface area of the ZnO/MXene composite provided more catalytic sites for MB, further improving the photocatalytic performance. The obtained composite photocatalyst exhibited high cycling stability, with the photocatalytic performance not significantly decreasing after four consecutive degradation cycles. Therefore, this work can provide a promising photocatalyst with high performance to deal with organic pollution in water.

## Figures and Tables

**Table 1:** Comparison of photocatalytic degradation activity of different photocatalysts for organic dyes.

Photocatalysts	Degradation Ratio (%)	Irradiation Time (min)	Performance Improvement	Light Sources	Ref
ZnO/V_2_C	99.8	120	17.8	500 W Xenon lamp	This work
ZnO/Ag	92.9	210	3	Sunlight	[40]
ZnO/Ce	94.68	60	9.1	300 W visible lamp	[41]
ZnO/Cu	91	75	1.62	30 W 254 nm-lamp	[42]
ZnO/rGO	99	120	12.03	300 W Xenon lamp	[43]
ZnO/Ti_3_C_2_	84	60	1.5	40 W 365 nm-lamp	[44]
ZnO/V_2_C	98.93	120	14.27	500 W Xenon lamp	[30]
ZnO/Nb_2_C	62.62	120	2.92	300 W Xenon lamp	[37]
ZnO/Ti_2_C	99.16	120	14.76	300 W Xenon lamp	[45]

## Data Availability

Data are contained within the article.
